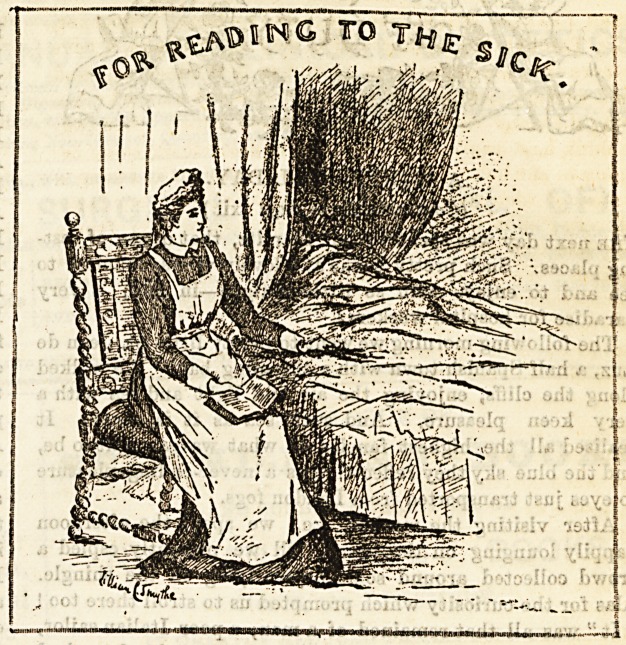# Extra Supplement—The Nursing Mirror

**Published:** 1890-12-27

**Authors:** 


Being the Extra Nursing Supplement of "The Hospital" Newspaper.

All Contributions for this Supplement should be addressed to the Editor, The Hospital, 140, Strand, London, W.C., and should have the word "Nursing" plainly written in left-hand top corner of the envelope.

### En Passant.

A MERRY CHRISTMAS.—To all our readers, we heartily wish a happy Christmas! First of all to our sisters and nurses, who are working in hospital wards, or in the private sick-room, we send our good wishes and our sympathy. Many of them may be deprived of family gatherings, the country dance, and children's games of a good old-fashioned Christmas, but we hope the hearts of all may be cheerful in spite of sad surroundings. In many hospitals the nurses have worked hard with decorations, theatricals, carols, and other festivities to amuse the sick; they will surely get their reward if Christmas Day can be made in any way one of extra brightness for their patients. It is no easy thing to keep Christmas properly in hospitals and infirmaries, and our admiration is given to those who, in pursuit of their vocation, have to work hard while others play. But if to all Christmas Day cannot be a holiday, it can be, what is far better, a holy day.

SHORT ITEMS.—The Marchioness of Breadalbane opened a very successful sale of work which has been held at Glasgow in aid of the Training Home for Nurses in Renfrew Street.—A nurse is wanted at the Greek Hospital, Alexandria; there is no matron, and the nurse works entirely under the doctor.—The Rev. H. B. Chapman says he has received a long letter from Sister Rose Gertrude, setting forth her reasons for abandoning the charge of the Leper Hospital, but that he cannot make the letter public.—The Bedford Trained Nurses Institute is in very low water just now, and a Committee of ladies have been appointed to try and raise necessary funds and increase the interest of the public in the Institute.—Leicester Institute of Trained Nurses makes an appeal for help at this season; at St. Martin's Church Spohr's "Last Judgment" has been given at two special services in aid of the Institute.

A MANCHESTER MEETING.—Mr. Oliver Heywood last week presided at the twenty-fifth annual meeting of the Manchester and Salford Sick Poor and Private Nursing Institution. The annual report, read by Dr. Donald, stated that a distinct advance had been made in the work of the charity during the year. With the establishment of a third home for district nurses the scheme which the Committee had had in view for some years had been completed, and lines definitely laid down on which the work of the institution could be most efficiently carried on. Manchester and Salford were now to a large extent covered by the institution, and every district nurse (excluding those who work in the outlying districts) lived in one or other of the district homes. The most important changes during the past year might be summarised by stating that a new district (Cornbrook) had been added, and that the nurses of the Medlock Street and Chester Road districts have been transferred to the Hulme Home. There had been some re-distribution of the districts among the various homes, and there were now 23 district nurses employed by the Institution, but an additional nurse had been engaged for Hulme, and would enter on her duties shortly, so that by the end of this year there would be 24 district nurses at work. The total number of cases visited during the year amongst the sick poor was 4,349, and the total number of visits paid to these cases was 81,197, being an average of about 18 visits to each case. The financial statement, read by Mr. R. B. Longbridge, on the General Fund showed a total receipt of £2,008 16s. 4d., which, after allowing for the necessary disbursements, left a balance in hand of £71 19s. 7d. The Rev. J. J. Twist moved a resolution affirming that the Institution was deserving of the increased support of the public. Such a plea, he said, was from his own knowledge of the good work that had been done in the homes of the sick poor fully justified, and he not only moved but supported it heartily. The resolution was seconded by Mr. Herbert Phillips and carried unanimously.

QUEEN VICTORIA NURSES.—The second annual meeting of the Scottish branch of the Queen Victoria's Jubilee Institute for Nurses was held in the saloon of the Royal Hotel, Edinburgh. The Lord Provost of Edinburgh presided, and in his opening speech made reference to the great loss the institute had suffered by the sad death of Lady Rosebery. It was announced that the Queen had appointed Princess Louise President of the institution, and that Miss Guthrie Wright, the able secretary, had been appointed an extra member of the Council. At present the Council are responsible for fourteen nurses. Only one nurse had been placed when the first report was issued. Since then four nurses had been withdrawn or not retained at the end of the month of trial; one nurse was withdrawn from the state of her health; seven nurses were placed during the year; eight in the district training home; two in the Maternity Hospital; and four in the Infirmary. The report refers to the lectures which have been delivered in the Home, and to the extension of the system of local nursing association. Preliminary steps had been taken by the Glasgow Sick Poor Nursing Association for Iaffiliation with the nstitute. During the year, 735 cases have been nursed, and 18,273 visits paid.

THOSE PROS. AGAIN.—We send our sympathy to the governors of the Suffolk General Hospital, for the management of that institution has been attacked, and once more the charges are brought by a six months' probationer. "Sarah Drury" writes: At tea the nurses were only allowed bread and butter and tea; now and then we had a small plain cake and once jam. Often the bread was so dry that we did not care to eat it, and several times we bought a new loaf with our own pocket money. Once I remember there was a piece of bread four days old, as dry as a chip, but at the same time there was another piece not so dry. At supper we generally had any meat which was left from dinner, and once or twice there was such a little, and that so fat and untempting, that most of us left it, and ate strong American cheese and bread, which was generally on the table. After complaining of the nurses' food Sarah Drury proceeds to complain of the patients. Truly the above menu does not strike us as unsatisfactory, and the occasional cake and jam for tea is more than most hospitals can afford to give their nurses. The Matron of the hospital has written the following reply: "I observe in the *Bury Free Press* of Saturday last a letter, published by Dr. Image, bearing the signature of 'Sarah Drury.' Had the letter contained a true statement of the facts, then the matter should have been submitted to the Committee, and not published to the world, as it has been with the inevitable result of doing great mischief to the work of the hospital, a result I should have imagined Dr. Image would have been anxious to avoid. The statements in Miss Drury's letter can be disproved, as no one knows better than Dr. Image, who had previously made similar charges to me in so violent and insulting a manner that I at once sent in my resignation to the Chairman of the Committee. On the following Tuesday the whole matter was inquired into by the Committee in the presence of Dr. Image, Miss Drury, and myself, with the result that I was absolved from any blame, and Dr. Image tendered his resignation. I should add that before the meeting Dr. Image had sent me an apology for what he had said. One word as to Miss Drury. She was a probationer here for six months. The night before she left the hospital she came to my room and thanked me for what she called my kindness to her, and said how very happy and contented she had been here. I need not trouble you further with regard to Miss Drury's cruel and groundless charges. I am ready to answer them in detail, and shall demand an investigation of the food supplied to patients and nurses."

### A Nurses' Christmas Box—£10,000.

It is with a feeling of deep and sincere thankfulness that we are able to publish the letters immediately following, and to announce that in addition to the Bonus Fund of £40,000, which already stood in the name of the nurses of this country, a further sum of £10,000 is now at their service in the shape of a Benevolent Fund, making in all £50,000. Those who are familiar with the history of the Royal National Pension Fund for Nurses, will hardly need to be told who it is that has once more come forward with the princely sum of nearly £8,000 to complete the efforts of the nurses themselves to raise a Benevolent Fund of £10,000 for the benefit of their disabled and helpless sisters. It is the family of the late Mr. Junius S. Morgan who have thus added a noble top-stone to a benevolent work which already seemed in its beautiful generosity more than complete. The late Mr. Junius S. Morgan, as one of many acts of beneficence, gave, before he died, £10,000 to the Royal National Pension Fund. Of several princely donors he was the chief. The nurses of Great Britain had come to know him as their trusted friend. To most of them he was but a name. But they had learnt to love his name. Although the idea of the Royal National Pension Fund arose in the mind of Mr. Burdett, that idea could never have become a concrete reality without the princely help of Mr. Junius Morgan, and the other city merchants whose names have so often appeared in these pages. Full of years, but all too soon, Mr. Morgan died. Nurses in every part of the country felt by a common instinct that a memorial should be raised to the memory of so good a man. They did not delay to put their hands to the work. It was decided that the memorial should take the form of a Benevolent Fund, for the purpose of helping those nurses who are in such unhappy circumstances that they can neither earn their own living for the present nor provide pensions for the future.

It is to be said to the honour of the nurses, that though they are a small body, and receive remuneration for their services of the most modest kind, they raised among themselves alone, for the help of their poorer sisters, no less a sum than £2,268 4s. 7d. This fund so raised, the family of the late Mr. Morgan, with a generosity like his own, have now increased to £10,000. The sum of £7,731 15s. 5d. was needed for this purpose, and it has been given by the members of Mr. Morgan's family in the following proportions, viz., J. Pierpont Morgan, £5,000; Sarah S. Morgan, £1,000; Mary L. Burns, £1,000; Juliet P. Morgan, £731 15s. 5d. It cannot but be felt, by nurses most of all, but also by medical men, hospital managers, and the public at large, that Mr. Morgan and his family in thus of themselves giving to the nurses of this country a sum of nearly £18,000, have done a deed of benevolence which has few parallels, and which will constitute an enduring monument to their name. The Fund is to be called the "Junius S. Morgan Benevolent Fund," and we do not know of any memorial which can better preserve the memory of a good man in the hearts of those whom his wealth has blest. The £10,000 thus given, and available for the nurses of this country, added to the £40,000, the last of which was given on Saturday, 20th inst., places them in a position of security and prospective comfort such as, five years ago, they neither hoped nor even dreamt of. Well, may all the donors and subscribers to this fund, and still more its original founder, feel that the day of the completion of their work has come, and that the work is both great, and useful, and good.

### THE FOLLOWING LETTERS HAVE BEEN SENT TO THE DAILY PAPERS:

Sir,—In January, 1888, I had the pleasure to announce in your columns that four City merchants (Lord Rothschild, Messrs. Antony Gibbs and Sons, E. A. Hambro, and Junius S. Morgan) had generously deposited £20,000 with the Accountant-General in Chancery to enable adequate provision to be made on a provident basis for all nurses by the establishment of the National Pension Fund. Later it was my privilege to report that this money had been made over, with an additional £5,000 from Mr. Morgan, as a free gift to the Fund. Subsequently it was determined to endeavour to raise the Donation Bonus Fund to £40,000.

Mr. Junius S. Morgan was from the outset the life and soul of the movement, in which he took the greatest personal interest. There was a widespread feeling of regret and sorrow at the time of his death, in April last, and the nurses throughout the country spontaneously expressed a desire to commemorate his memory by establishing a benevolent branch of the Pension Fund, to be called the Junius S. Morgan Benevolent Fund. Several hundred purses were accordingly presented to Her Royal Highness the Princess of Wales at Marlborough House on July 4th, 1890, on the occasion of the presentation by the Princess, as President of the Fund, of certificates to the first thousand nurses. These purses contained £2,268, which the Council have vested in the hands of three trustees (Mr. Walter H. Burns, Mr. Edward Rawlings, and another), and the income therefrom will be devoted to secure (1) the immediate pecuniary or other relief, by loan or absolute gift, of matrons, sisters, and nurses (if members of the National Pension Fund) who may be in distress, and to assist them in keeping up the payment of premiums on any policy they may have taken out in the society, and (2) annuities to matrons, sisters, and nurses who may have been unable to provide for themselves after sixty years of age. The trustees will be assisted in the distribution of the Benevolent Fund by a committee consisting of the Lady Patronesses, of representatives (a) of the policy holders and annuitants, (b) of the Council of the Fund. It must be manifest, and if adequate service was to be rendered, that the £2,268 could only form the nucleus of a much larger fund. I have therefore great pleasure in asking you to publish the enclosed letter from Mr. Burns, as the representative of the family of Mr. Junius S. Morgan, intimating that they have determined to raise the funds of the Morgan Memorial Benevolent Fund Trust to £10,000. I am sure these splendid gifts will be gratefully welcomed by everybody.

The Donation Bonus Fund now amounts to upwards of £40,000, and the whole of the £50,000 has been obtained without practically any of the usual expenditure on printing, stationery, postage, etc. In other words, the money has been spontaneously subscribed by those interested in nurses, as a mark of their appreciation of the services rendered by the noble women who pursue this arduous calling, often at the risk of their lives, and always with devotion and conscientiousness.—I am, etc., HENRY C. BURDETT.

The following is the letter referred to :—

22, Old Broad Street, E.C.,
18th December, 1890.

Dear Mr. Burdett,—The family of the late Mr. Junius S. Morgan were deeply touched by the spontaneous tribute paid to his memory by the nurses belonging to the National Pension Fund, in collecting among themselves the sum of £2,268 4s. 7d., and establishing therewith a benevolent fund in connection with that institution. His children desire to supplement and increase this sum—now called the Junius S. Morgan Benevolent Fund—from its present amount up to £10,000, and for that purpose have handed to me the sum of £7,731 15s. 5d., in the following proportions, viz. :—

| | | | |
|---|---|---|---|
| J. Pierpont Morgan ... ... ... | £5,000 | 0 | 0 |
| Sarah S. Morgan ... ... ... | 1,000 | 0 | 0 |
| Mary L. Burns ... ... ... ... | 1,000 | 0 | 0 |
| Juliet P. Morgan ... ... ... | 731 | 15 | 5 |
| | 7,731 | 15 | 5 |
| Making with the amount already subscribed by nurses, viz. ... ... | 2,268 | 4 | 7 |
| | £10,000 | 0 | 0 |

I have great pleasure in advising you and the Council of the National Pension Fund for Nurses of this donation, and beg to state that I hold the money at the disposal of the trustees of the Junius S. Morgan Benevolent Fund.—I remain, dear sir, yours sincerely,

(Signed) WALTER H. BURNS.

H. C. Burdett, Esq., Founder National Pension Fund.

### Everybody's Opinion.

*[Correspondence on all subjects is invited, but we cannot in any way be responsible for the opinions expressed by our correspondents. No communications can be entertained if the name and address of the correspondent is not given, or unless one side of the paper only be written on.]*

### AN APPEAL.

"F. T." writes from the Hertford Hospital: Will you allow me to plead in your columns for a nurse who has been married but a few months, and whose husband has lost his employment through no fault of his? She can make none of the preparations so dear to a woman's heart for the inevitable baby, which is expected to arrive in the spring; and I thought, perhaps, some matron, who no longer requires her stock of baby linen, would make a parcel, and send it to Miss Ritchie, 20, Avenue Carnot, Paris, a friend who would receive it for her. It goes to my heart to think of the baby's destitution, when it opens its eyes on this sad world. The postage of a good-sized parcel is only one and fourpence, and I would thank any one most heartily in the name of the poor little stranger.

### MALE NURSES.

"E. H. A." writes: May I be allowed to say a few words in favour of male nurses? After having had three nurses for my dear husband, I found for some time he was almost more than a woman could manage, so I wrote to the Hamilton Association for a male nurse; they supplied me with an excellent one, kind and gentle as a woman. He had great tact, with firmness; in fact, I cannot speak too highly of him. I was a nurse myself for many years, and know from personal experience many a gentleman (in private nursing) would much prefer a male nurse to do various things for them which now is undertaken by a female nurse. I would suggest that men be trained at such hospitals as St. Peter's for Stone, St. Mark's for Fistula, and many others; but I do not see myself, with proper regulations, why they should not be trained in a general hospital. Another small item (but not so to me) for which I was most grateful to the Hamilton Association was that they took half fee, as my dear husband was a doctor. No other institution from which I had nurses would make a reduction, not even his own hospital, where he had worked all his life until he broke down.

### A WEDDING GIFT.

"E. B. B." writes: I am charmed with Miss Durham's suggestion of a wedding gift for Princess Louise Augusta, and will gladly help towards it, for I know well how Princess Christian has worked for nurses. I think the Princess would be better pleased if the gift were of small intrinsic value, so I would suggest that nurses be invited to contribute any sum from 6d. upwards. Will Miss Durham undertake to receive the subscriptions?

### LADIES' COMMITTEES.

"NURSE" writes: I do not often write about anything which is published in THE HOSPITAL, because, in the first place, I have little time, and, in the second, by the exercise of a little patience, I generally find what I would say said by a much better writer than I am. But on the subject of ladies on the committee, I think I know something about the matter. I have worked under a Ladies' Committee for the last six years, and am quite content with my lot. Let me, however, say that they are ladies, and behave as such. I am nurse matron of a cottage hospital containing twelve beds, and generally full. The only resident, besides myself, is a general servant. I have little time, and less inclination, for visiting. I frequently do not go outside the gates for weeks together, and very lonely my life would be were it not for the ladies, who visit, assist with the accounts, bring flowers, and cheer and brighten us up generally. There are two committees, a ladies' and a gentlemen's; each meet once a month, and meet together once in three months. The ladies take it in turns to visit, one each week. Of course, they are not all alike, and it is more pleasure to see some than others; some are also more practical than others. Still, if asked, I should say please leave me the ladies' committee.

### Death in Our Ranks.

ON December 9th, at 11, Hunby Road, Tooting, Nurse Annie (Rae) Bishop, aged 24. While doing relief work at Paddington Green Children's Hospital, Nurse Bishop contracted typhoid; she returned to her home to be nursed, but only lived three weeks. Her gentle loving ways had endeared her to friends and patients both near and far.

From Melbourne we hear with regret the death of Mrs. Harriet White Wylie, for 21 years matron of the Victorian Eye and Ear Hospital. She was greatly and deservedly respected, and worked up to the last, regardless of her own sufferings. She was the widow of the late Captain William Wylie.

### Notes and Queries.

TO CORRESPONDENTS.—1. Questions may be written on post-cards. 2. Advertisements in disguise are inadmissible. 3. In answering a query please quote the number. 4. If a private answer is desired, a stamped addressed envelope must be forwarded.

### Answers.

*M. E. C.*—We only insert items of general interest.

*E. Murray.*—The Rural Nursing Association. Write for particulars to Mrs. Mallison, Dixton Manor, Winchcombe, Gloucestershire.

(20) *Percentage.*—The following is the rate given to the private nurses of the Hospital for Sick Children, Great Ormond Street: Fixed wages—First year, £25, and no percentage; second year, £25, and 10 per cent. on the sums received for the nurse's services; third year, £25, and 15 per cent. on the sums received for the nurse's services; fourth year, £25, and 20 per cent. on the sums received for the nurse's services; fifth year and after, £25, and 25 per cent. on the sums received for the nurse's services.

*J. W.*—"Poor People's Christmas" is published by Elkin Mathews, of Vigo Street. Price 6d.

### Christmas at Vancouver.

Sister Frances writes from St. Luke's Home, Vancouver, B.C.: Through the kindness of a lady in England, I have for more than a year been in receipt of The Hospital, and now I venture to write and ask you to make us a little known among the many readers of your valuable paper. You cannot think how eagerly we read and appreciate anything belonging to our beloved nursing, besides occasionally seeing a the name of some nurse we know, and we look for your paper with very glad feelings. St. Luke's Home has now been of three years' standing, and is very generally known to visitors from England. We are only a small staff, two trained nurses and a probationer, and with occasional help from patients or ladies in need of a temporary domicile we work the Home. We have seven beds upstairs, and a large ward with six beds downstairs, two small bed-rooms, dining-room, and reception-room comprise our Home. Sometimes we have all women upstairs; this we generally contrive, but if there is a serious male case who must be in a private room, he has to be taken upstairs, and at present we are obliged to just use our rooms as our means and applications allow. The indoor work is our first duty, but often we are with just one patient; then we go among the poor and generally help all around; then again we offer a bed to any traveller; young girls, passing through, often come and ask me what to do or where to go. We enquire into their circumstances and help them, either taking a small sum from them for their board while in the "Home," or expecting a few hours' help each day in the general work, while they are suiting themselves as to employment, or while communicating with their friends. The clergy send such cases to us frequently, and we meet the train and try to help protéges so sent, governesses, nurses, lady helps, all pass in the course of the year through our hands. Three years is a long time to be resident here, so we are looked upon as "old timers;" consequently the other old timers look upon the Home as their peculiar property, and in sickness, or should death occur, we are at once sought for, with the usual apology, "Sister, I am so sorry, but you know us, &c." So we see all the sad side of life; and now, alas, we are beginning a new phase—genteel poverty. Each winter seems to make this worse, and now I come to my reason for writing and wanting to become known. What I should like is interest centred in us among the nursing profession, and if individually we could hear now and then from our mother country and our nursing sisters, it would lighten our labours, and I think, too, without much monetary loss, we might be very substantially helped. I mean, a nurse has little spare money, but she has unbounded interest; used in a right spirit, such missionary work as ours could be furnished with necessaries that we can never hope to obtain. Again, among the mass of nurses they have friends and relations continually coming through. Hospital necessaries such as thermometers, medicine spoons, or rubber goods, in fact, many things made into small parcels would be only too thankfully received by us.

Perhaps it will interest you to know how we spend Christmas. We get ready all these weeks collecting clothing, money, and food, and on Christmas Eve we get all our dependants and give everything away. Then we arrange our meals according to our means; we lay breakfast in the large ward which we have hitherto had empty and given up for that day; then we all make our communion in our little church, which is next door, then we come back, and by eight o'clock are ready for all and any from a distance who come to church for the eight o'clock service. We have a very plain breakfast—bread and butter, marmalade, and coffee—and about a dozen avail themselves; then we search through our treasures, and provide cards and gifts for patients—little children and home-sick new-comers—and although very home-sick ourselves, we begin to find Christmas Day actually merry. Then we divide the morning, and half go to church at eleven a.m., and after that we sit down to a lunch, at which our rector presides, and this is the beginning of our day. He brings in all the solitary young men, so we never know how many we shall be. Patients and all sit together; and then the event of the day comes—five o'clock dinner; this is the rector's own, and he invites his guests, who are all men, and who otherwise would dine alone at their hotels. We generally sit down thirty and we are just a genuine English home party and we contrive to have our same home dinner; then we all help to clear away, and we spend the evening in games, and so ends to one and all a very happy day. Then comes our next great event, New Year's Eve. We give a general invitation to patients, helpers, nurses, clergy, choir, and all associated with us during the past year, our friends help us with the "supper" and we sit down to high tea at eight o'clock, and end up with midnight service in our Church. This party is perhaps the most looked forward to, for it embraces high and low, rich and poor, and to many it is their only outing; so we do our very best to make this a success, and we generally find our new made Christmas dinner guests only too glad to offer their services in helping us entertain and prepare for our party, and to this your readers could help us We would be glad of letters, pictures, cards, games, any amusing book, or any hints as to new games. Our Christmas and New Years are matters of great importance and give us lots of preparing, for we are bound to keep things going and be a success.

This year we shall give our New Year's party in the school-room. It will be the anniversary of the death of one of our number, and, alas, the absence of many of our former friends. Except the rector and myself, all our indoor help will be new, so, too, with the clergy; we have quite a gap, for the diocese has lost several who have chosen other and more dangerous fields of mission work. Now, I think my account is getting rather lengthy, so I will conclude by giving a description of our present household, and who will be with us for some time. Sister Gertrude, the head nurse, is a widow, her little boy, four years old, is here, a very delicate child, and who has been with us from the beginning and left in our care while his mother went through a two years' hospital training and then gave her work to us; the probationer Nurse Rhoda is a young English girl, 19. We have a naval officer's little girl, paralyzed, ten years old. She was playing with some children one day and fell heavily on her knees. In three days she could not move, and now she is helpless. We have a destitute case in for confinement, a young widow, 19, and now, with myself, you have our regular inmates and those who know no other home. One thing more. We took great interest in the account of the doll show, and only for a severe strain of work we should have sent ourselves in miniature. We wish you and your many readers a very merry Christmas and a happy New Year.

### Presentation.

The London Hospital.—On December 16th the staff nurses and probationers of this hospital presented their matron, Miss Luckes, with a beautiful little silver coffee-pot and a bouquet of flowers. The gift was accompanied by the following letter.—"It will add not a little to the bright and happy Christmas we always spend here, if you will accept this coffee pot and these few flowers as a slight token of our gratitude to you for your work for us in the past, and a pledge of our banding ourselves together, to help you in the future by being earnest and loyal workers."

### A Christmas Legend.

Time was when reigned a certain King, whose fame
For playful wisdom has outlived his name,
A King who, ruling seldom with the rod,
Guided his people gladsomely to God.

Close upon Candlemas, one happy spring,
When court and subjects gathered round the King,
He proclamation made, by royal command,
Which stirred an impulse throughout all the land.

"Know by these presents"—spake the sovereign will—
"Whoso by Christmastide shall best fulfil
Our goodly purpose, he shall guerdon bear
Of golden treasure, and our favour share.

"Whoso with most success shall kindly rear
What brightest is, and best, within the year,
What we may judge the purest, whitest thing,
He shall be named the victor by the King."

Such was the edict; and ambition then
Began to occupy the minds of men;
Each striving, in his rank, by healthful ways,
To cherish what might win him highest praise.

So the realm prospered—homestead, field, and fold—
As months passed on, through hope of promised gold
For him who finally the prize should bring
Of truest spotlessness, to please the King.

Then Christmas came, while yet the land was green,
And lingering tints of verdure still were seen;
A bracing spell of sun, and sparkling rime,
Ere bleakest winter had its hardest time.

And now, at noon, on the great Noel day,
The choicest claimants from the large array
Of all who thronged to seek the King's award
Stood, proudly eager, in the Palace yard.

A simple workman, who, with loving pains,
Had lavished on a flower his little gains
Week after week, now laid a lily sweet,
With pearly petals, at his monarch's feet;

A gentle youth, whose soul was set above
Mere earthly scholarship, had fed a dove,
Which, stainless, and unsullied from the nest,
He reverent placed within the monarch's breast;

A sturdy yeoman, big with fond desire
To serve his lord, had fostered in the byre
A milk-white heifer, which, superbly grown,
He led with triumph to the monarch's throne;

A stately squire, with his well-favoured dame,
To bring their modest, meek-eyed daughter came:
Than whom no maid, of summers seventeen,
More fair and faultless, waited on the Queen.

Of heifer, dove, sweet flower, and maiden fair,

In pure, white contrast to the trim parterre

Of the quadrangle, as the sunbeams fell,

Which seemed most spotless it were hard to tell!

Therefore the King, who held a wise intent,
His gaze on each in turn uncertain bent,
Then bade them all another week to bide,
Till New Year's Day the contest should decide.

But, as the week pursued its onward course,
Keen winds brought snow, in fast and constant force,
So that the kingdom with a mantle white,
And dazzling was on New Year's morning dight:

And when again the candidates were ranged
Around the King, each bore an aspect changed
From former excellence; the lily's hue
Was to its pristine splendour scarcely true;

The dove's soft plumage, which so chaste had shown,

Betrayed a look as if less lustrous grown;

Whilst from the snow the fierce reflected gleam

Made the white heifer saffron-tainted seem;

Even the clear translucency of face

Which lately lent the maiden classic grace,

Disclosed some subtle blemishes to sight

When tried by such severity of light.

Then did the monarch publicly avow

His sage decision, hidden until now,

Which, by his heralds, with a trumpet's blast,

He thus delivered to the concourse vast:

"The 'Lily-bearer'—this is our decree—

Shall by the State henceforth supported be;

And, for the gentle scholar, with his dove,

A yearly pension we hereby approve.

"The trusty yeoman, with his heart so warm,

Shall be our bailiff on the Palace-farm;

And the sweet maiden, at her parents' will,

A post of honour to the Queen shall fill.

"But we bid each take notice—with the rest

Of all our faithful subjects—that the best

And brightest things our kingdom could supply,

Failed when God's snow came down with them to vie.

"So let our land the golden lesson learn

That for our purest pleasures we must turn

To heavenly sources: where we humbly know

Our 'sins of scarlet' are made 'white as snow.'"

W. T. FERNIE.

### CHRIST IN OUR HEARTS.

Long centuries have passed away. The world has rolled forward through numberless changes since the day broke which give a Saviour to the earth. Time moves, but eternity stands still, and we, amidst all the changes of the former, can rest in faith and certain hope of eternal life.

And do not our hearts rejoice within us as again the happy time of Christmas comes round? The sweet strain of the angels sounds in our ears, "Peace on earth, goodwill towards men," and we echo back the enchanting anthem, "Glory to God in the highest, glory to God on high."

And these comfortable words meant, and still mean, the same thing for all men, and yet different things for each one in particular, according to his needs; for Christ has come, and if we are weary He will refresh us, if we are captives to sin He will set us free, if we are blind to the mercies God sends us He will dispel the darkness from our eyes.

How beautiful are all infants in the light of this "Babe of Bethlehem!" They catch the reflexion of His purity and guilelessness, and we also may rest in His love, if we become meek and lowly followers of Christ. The child in the manger is King, and we will worship Him with heart and voice, with our gold if we have any, with the incense of our prayers and good actions, and the myrrh of love, and warmth and charity to all men in our hearts. The world would be fragrant indeed if we all did our parts; let us try for one day at least to make it so. We may be poor and obscure like Joseph the Carpenter, yet we have Christ to protect in the form of His little ones. Our homes may be lowly and humble as the stable at Bethlehem, but Christ has promised to take up His abode with the humble and contrite spirit. Christ hath exalted the humble and meek. Then:

O come, all ye faithful,
Joyful and triumphant,
O come, let us adore Him, Christ the Lord.

O ye heights of heaven, adore Him,
Angel-hosts His praises sing;
All dominions bow before Him,
And extol our God and King.
Let no tongue on earth be silent,
Every voice in concert ring,
Evermore and evermore.

### A WINTER HOLIDAY.

(*Continued from page* lxii.)

The next day was spent in fair Biarritz, that queen of resting places. Such pretty walks and lovely views, so much to see and to enjoy, with so little fatigue—in fact, a very paradise for hospital workers!

The following morning we went to pretty little St. Jean de Luz, a half Spanish town with a charming bay. We walked along the cliffs, enjoying the air and light and sea with a very keen pleasure. And such sea as it is there! It realised all the highest fancies of what waves ought to be, and the blue sky they reflected was a never-ending pleasure to eyes just transported from London fogs.

After visiting the market, &c., we spent the afternoon happily lounging on the beach, till we presently espied a crowd collected around some object lying on the shingle. Alas for the curiosity which prompted us to stroll there too! "It" was all that remained of a man, a poor Italian sailor, who, with several others and a poor little cabin-boy, had been drowned a few days before. Their ship had struck on a rock in the bay, and they had perished in sight of shore, before any assistance could reach them. The other bodies had been already recovered, but this ghastly caricature of "the noblest work of God" had but just been washed ashore.

Who was waiting, in sunny Italy, wife, child, mother, or sweetheart, for "he who would never come back to the town?"

A cloud came over our bright afternoon, and our careless enjoyment was arrested. We walked back to the station in silence.

Our next day's expedition was full of interest, for we crossed the Spanish frontier and reached San Sebastian of historic fame.

Passing the Bull ring, we began our way to the fortress by ascending a rather steep hill, which we presently discovered was a rock nearly surrounded by the sea. When I say "sea," I pray you do not think of Brighton or Lowestoft, but picture first a brilliant blue sky, and below that the great, strong, splashing, dashing waves, "mountains high," which make a never-ceasing roar as they thunder below "the everlasting hills" on which this wonderful fortress stands, an evidence of man's pride, reared on one of Nature's masterpieces!

We wandered on at our leisure, drinking in fresh life and vigour with each clear, sweet breeze. We found on our way some damp little crevices, and peering curiously within, found fronds of the dainty maidenhair fern growing unharmed, though scarcely sheltered from the salt sea spray. Only parts of the fortress are shown to strangers, but the view from the walls would have well repaid a longer walk.

When we descended to the town again, we still found plenty to interest us. Churches, harbour, shops, and gardens, and before leaving we saw, to our amusement, the old-world little town was illuminated with electric light. A funny little incident occurred at the harbour where we rested awhile on an upturned boat.

A group of handsome, dark-eyed children surrounded us, and seemed somewhat puzzled by our inability to understand their *patois*. Presently one bolder than the rest, came up and stroked a shawl which Frances carried, and taking a bit of the ball fringe which edged it, she put the woolly thing into her mouth. Apparently disgusted at the uninviting flavour, she coolly wrenched it off and threw it into the sea. For this piece of impertinence she was evidently hugely admired by her companions, several of whom escorted us to the station, where we found our train ready to return.

(*To be continued.*)

### New Competition.

Bed-jackets for competition were received from F. E. Margaret Hart, Elinor May Lucas, E. Hardy, Miriam Payne, Mary Wood, Sarah A. Whitbread, Nurse Bartlett, Nurse A. Perry, Marion and Violet Black, Nurse Owery, Nurse Hutton, Nurse Steer, E. M. Cook, Ethel Heachan, Mrs. Cargill, Amy Anderson, "Jenny Wren," L. E. Pountney, Mrs. Ramsay, Kate Prior, Ellen Tarrant, A. E. Moore, Sister K. Pitts A. L. Strong, and Sister Chadwick, Ellen Tryer, Nurse Messiter, Miss Barbara A. Summers, Miss McRae, Nurse F. L. Saunders, "One who dearly loves hospitals," Nurse Jane Macdonald, Nurse Fernie, Nurse E. Maystone, H. E. Fuller, Ellen Dudley, and Nurse Gerrard. We feel like writing a folio volume treatise on bed-jackets after a careful inspection of all those garments sent in by the above ladies. Who, though, could describe the beautiful embroidery on the pink nightingale with hood sent by Mrs. Cargill, matron at Aldershot? Difficult also to describe is the boy's jacket made of blanket from Nurse Messiter; thick, warm, and comfortable, the motto evidently being "use before beauty"; just the sort of thing to charm a boy, because there is little that is feminine about it save the exquisite needlework. Then Nurse Oswestry had sent a jacket buttoning across the front, a quite original shape; and Nurse Tarrant, Nurse Prior, and others have used elastic for the sleeves (top and bottom) and for the neck. A pretty cape with inside half-sleeves, like mantle sleeves, was sent by Miss Hart; Miss Pountney sent a nice cape; indeed, some of the capes were especially good. A large number of jackets fastened both back and front, and also the sleeves buttoned from top to bottom. Nurse Hardy has put some excellent needlework into a comfortable blue flannel jacket; Nurse Maystone has put a sensible full front into an exquisitely made jacket, fastening behind. In ingenuity, Miss Whitebread, Nurse Moore, Miss Chadwick, and Miss Bartlett excelled, their jackets simply opened all over! They were eminently fitted for surgical cases. Worthy of honourable mention are the neat and useful jackets sent by Miss Anderson, Nurse Summers, and Nurse Saunders. After much consideration the First Prize is awarded to Nurse Ethel Heachan, and the Second Prize to Nurse Ellen Tarrant. We shall be glad to hear from these nurses if they prefer their prizes in books or money. The kind thought which made many nurses include handkerchiefs, Christmas cards, &c., will, we are sure, be appreciated by the recipients of the jackets. The story of the distribution will be told next week.

### Amusements and Relaxation.

**N.B.—THIRD QUARTERLY WORD COMPETITION**

**Commenced Oct. 4, 1890; ends Dec. 27, 1890.**

Three prizes of 15s., 10s., 5s., will be given for the largest number of words derived from the words set for dissection.

N.B.—Word dissections must be sent in WEEKLY not later than the first post on Thursday to the *Prize Editor*, 140, Strand, W.C., arranged alphabetically, with correct total affixed.

The word for dissection for this. the THIRTEENTH week of the quarter, being "SKATING"

| Names. | Dec. 18th. | Totals. | Names. | Dec. 18th. | Totals. |
|---|---|---|---|---|---|
| Jenny Wren | 24 | 646 | Checkmate | — | 76 |
| Tinie | — | 55 | Silver King | — | 163 |
| Agamemnon | 24 | 664 | S. Anthony | — | 76 |
| Patience | 24 | 663 | Quackah | — | 75 |
| Ecila | 22 | 662 | Reynard | — | — |
| Lightowlers | 22 | 634 | Sally | — | 27 |
| Rouge | — | 89 | Success | — | 61 |
| Wyamaris | 24 | 654 | Caledonia | — | 52 |
| Qu'appelle | 22 | 561 | Nurse Emma | — | 305 |
| Nosam | — | — | Hazel | — | 29 |
| Nurse Hilda | — | 44 | Pallas | — | 48 |
| Lady Betty | 23 | 609 | Puss | — | 15 |
| Grenelle | — | 43 | Shakespeare | 20 | 545 |
| Daisy | — | 324 | Melita | — | — |
| H. A. S. | — | 157 | Nora | — | 10 |
| A. B. C. | — | 66 | Elsie | — | 35 |
| Liz | — | — | Espèrance | — | 27 |

SPECIAL NOTICE TO CORRESPONDENTS.

New quarterly word competition commences January 3rd, 1891.

## Figures and Tables

**Figure f1:**